# An outbreak of acute haemorrhagic conjunctivitis associated with coxsackievirus A24 variant in The Gambia, West Africa

**DOI:** 10.1186/s13104-017-3007-9

**Published:** 2017-12-06

**Authors:** Sarah E. Burr, Ansumana Sillah, Hassan Joof, Robin L. Bailey, Martin J. Holland

**Affiliations:** 1 Clinical Research Department, London School of Hygiene and Tropical Medicine, London, UK; 2 Disease Control and Elimination Theme, Medical Research Council Unit, The Gambia, Fajara, Banjul, The Gambia; 3 National Eye Health Programe, Ministry of Health and Social Welfare, Kanifing, The Gambia

**Keywords:** Apollo 11 disease, Acute haemorrhagic conjunctivitis, Coxsackievirus A24 variant, The Gambia

## Abstract

**Objective:**

An outbreak of acute haemorrhagic conjunctivitis occurred in The Gambia, West Africa in 2011. Affected individuals presented with conjunctival haemorrhages, swelling and ocular discharge. In an effort to identify a causative agent of the disease, ocular swabs were taken from patients during the acute and convalescent phases. Total RNA was extracted from all samples and reverse-transcriptase PCR performed using primers specific for all enteroviruses. Resulting amplicons were sequenced and data compared to known sequences using the BLAST algorithm.

**Results:**

Forty-eight swabs were included in the analysis. Of these, 21 acute and 9 convalescent swabs (65% of the total) gave positive PCR results. Sequence analysis of the resulting amplicons indicated 99% sequence identity with coxsackievirus A24 variant identified during independent outbreaks of acute haemorrhagic conjunctivitis around the world and suggest the Gambian outbreak was due to this virus.

**Electronic supplementary material:**

The online version of this article (10.1186/s13104-017-3007-9) contains supplementary material, which is available to authorized users.

## Introduction

Acute haemorrhagic conjunctivitis (AHC) was first described in Ghana in 1969 [1]. Its appearance coincided with the first Apollo moon landing prompting the Ghanaians to give it the name Apollo 11 Disease [2]. The etiological agent in this outbreak was identified as enterovirus 70 (EV70) [2, 3]. In the following year, an outbreak of Apollo was described in Singapore [4] however, in this case, the cause was determined to be a coxsackievirus A24 variant (CVA24v) [5]. Since then numerous reports of AHC have emerged from across the world, associated with either EV70 or CVA24v. These two viruses are now recognized as the primary agents of AHC [6].

AHC is not uncommon in The Gambia, West Africa with large-scale outbreaks occurring every few years. This is likely due to the breakdown of heard immunity as levels of neutralizing antibodies steadily decrease in the years following infection [7]. AHC usually resolves without sequelae, although secondary corneal infection has been reported after treatment with topical steroids [8]. In The Gambia however, most individuals do not seek treatment for the disease within the health care system but rather self-administer traditional treatments than can include applying lemon juice, paste of chili peppers or urine to the eyes, often further exacerbating clinical signs of disease.

In August 2011, an outbreak of AHC began in The Gambia and rapidly spread across the country. Infected individuals presented with conjunctival haemorrhages, conjunctival swelling and ocular discharge. As no formal reporting structure for AHC exists in The Gambia and given the lack of laboratory facilities in the country, little else is know about the outbreak.

We hypothesized that individuals with clinical signs of AHC would have ocular infection with either EV70 or CVA24v. In an effort to determine if either of these viruses were the causative agent of the outbreak, ocular swabs were collected from individuals presenting with clinical signs of AHC during the acute phase of the disease and following a period of convalescence. These swabs were then processed using PCR and DNA sequencing techniques. This represents the first identification of a causative agent of AHC in The Gambia.

## Main text

Ocular swabs were collected by convenience sampling of patients, residing in the Brikama and Mansakonko Local Government Areas (previously known as Western and Lower River Divisions), who presented with clinical signs of AHC to community eye health workers during a community screening exercise in September 2011, during the height of the outbreak. Inclusion criteria were the ability to provide informed consent, or in the case of minors, a parent or guardian willing to provide consent. Exclusion criteria were a pre-existing eye condition that prevented sample collection. Follow-up swabs were then collected for the same patients in December 2011, during the convalescent period. A total of 92 swabs were collected however, due to logistical and financial constraints, only 28 swabs collected from patients during the acute phase of the disease and an additional 20 from patients who were re-visited 3 months later were analysed in this study.

The age range of patients for whom samples were analysed was from 7 months to 65 years (mean = 10 years). Forty percent of these patients were female. All patients had clinical signs of AHC at the time the first swab was collected. One patient also had clinical signs of trachomatous scarring. No individual had signs of AHC when the convalescent swabs were collected.

One field supervisor with dedicated eye-health training and over 20 years experience in community eye-health collected all samples. Samples were collected from the lower fornix using sterile, Dacron swabs (Puritan, Maine, USA) and placed into screw-top tubes containing RNAlater (Thermo Fisher Scientific, Waltham, USA). Samples were held on wet ice in the field and frozen at – 70 °C within 12 h of collection. RNA was extracted using the QIAamp Viral RNA mini kit (QIAGEN, Hilden, Germany) as per the manufacturer’s instructions. RNA was reverse-transcribed and the resulting DNA amplified by polymerase chain reaction (PCR) using the Quantitect Virus RT MasterMix (QIAGEN, Hilden, Germany) and primers S1 (5′ CAAGCACTTCTGTTTCCCCGG 3′) and AS-1 (5′ ATTGTCACCATAAGCAGCCA 3′), which are specific for enteroviruses [9]. A nested PCR reaction was then carried out using primers S2 (5′ TCCTCCGGCCCCTGAATGCG 3′) and AS-2 (5′ AAACACGGACACCCAAAGTA 3′), again specific for enteroviruses [9]. Resulting amplicons were separated by gel electrophoresis and visualized with ethidium bromide staining. Of 48 swabs assayed, 21 acute and 9 convalescent samples (65% of all samples) generated amplicons of expected size (approximately 120 base pairs). Positive and negative PCR controls gave the expected results. Fisher’s exact test revealed a trend suggesting acute swabs were more likely to yield a positive PCR result than convalescent swabs (p = 0.0681), however this did not reach statistical significance when using a threshold for significance of p < 0.05.

Amplicons were subsequently purified and sent to Macrogen Inc. (Seoul, Korea) for Sanger sequencing. Sequencing chromatograms were edited manually using Sequencher 4.9 software (Gene Codes Corporation, Ann Arbor, MI, USA) and sequences were trimmed such that the first 25 bases contained less than 5 bases with confidence scores below Q20. A 71 base pair consensus sequence, formed from data from 26 amplicons, was then constructed (Additional file 1). All samples revealed identical sequences except one, which had a single base pair substitution at position 38 (G instead of T).

**Fig. 1:**
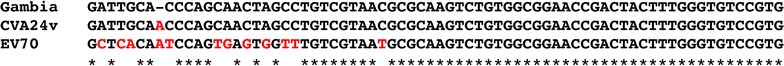
Multiple sequence alignment generated in T-Coffee [16]. Consensus sequence data obtained from ocular swabs from Gambian patients presenting with acute haemorrhagic conjunctivitis (AHC) in comparison to CVA24v (GenBank Accession No. KR399988) and EV70 (GenBank Accession No. D00820)

A comparison of the consensus sequence with known data was carried out using BLAST [10] and revealed 99% sequence identity with CVA24v associated with recent outbreaks of AHC in Reunion Island (Indian Ocean) in 2015 (GenBank Accession No. KR399988, positions 492–563) [11], Japan in 2011 (GenBank Accession No. AB769164, positions 496–567) [12], China in 2007 (GenBank Accession No. JF742579, positions 491–562) [13] and Singapore in 2005 (GenBank Accession No. DQ443002, positions 495–566) [14], all of which have been identified as belonging to genotype IV [11]. Eighty-three percent identity was found with EV70, spanning position 489–561 of the reference (GenBank Accession No. D00820) [15] (Fig. 1).

## Conclusions

Our data suggests that CVA24v was the causative agent of the outbreak of AHC, which occurred in The Gambia in 2011. Community ophthalmic nurses can use this knowledge to educate people as to the cause of AHC, to promote simple hygiene measures to interrupt transmission and to advise more appropriate means of clinical care in future outbreaks.

## Limitations

The greatest limitation of our study is the absence of swabs collected from individuals with normal eyes during the outbreak, which prevents us from saying CVA2a was the definitive cause of the AHC outbreak. That CVA24a was found in convalescent samples suggests the virus can persist in the eye for a significant period of time following the resolution of clinical signs, however, we have not found any published reports of CVA24a being detected in normal eyes outside of AHC outbreaks.

Twenty-one of 28 acute swabs (75%) generated a positive PCR result. The apparent absence of CVA24a DNA in the remaining 7 acute swabs may be due to the sensitivity of the test used, which was a nested PCR followed by visualization of amplified DNA by gel electrophoresis and ethidium bromide staining. It is possible that these 7 individuals were not yet shedding viral DNA at the time of sample collection or were shedding loads that were below the sensitivity of the test. Misdiagnosis of AHC by the eye-health worker who collected the swabs is also a possible explanation however we feel this is unlikely given the severity of clinical signs observed and the contagious nature of AHC.

## Additional file

**Additional file 1.** Consensus sequence. A 71 base pair consensus sequence, formed from data from 26 amplicons.

## Abbreviations

AHC: acute haemorrhagic conjunctivitis
CVA24v: coxsackievirus A24 variant
EV70: enterovirus 70
