# Supplementary material for: An outbreak of acute haemorrhagic conjunctivitis associated with coxsackievirus A24 variant in The Gambia, West Africa
Source: BMC Res Notes. 2017 Dec 6;10:692. doi: 10.1186/s13104-017-3007-9 (PMC5717804; doi:10.1186/s13104-017-3007-9)
Supplement: Supplementary file 1 — Additional file 1. Consensus sequence. A 71 base pair consensus sequence, formed from data from 26 amplicons. [file 13104_2017_3007_MOESM1_ESM.doc]

File name: Additional file 1

File format: .doc

File title: Consensus sequence

File description: A 71 base pair consensus sequence, formed from data from 26 amplicons.

GATTGCACCCAGCAACTAGCCTGTCGTAACGCGCAAGTCTGTGGCGGAACCGACTACTTTGGGTGTCCGTG
